# The contribution of dietary composition over 25 years to cardiovascular risk factors in childhood and adulthood: the Princeton Lipid Research Study

**DOI:** 10.1017/S0007114524001521

**Published:** 2024-09-14

**Authors:** Leah C. Beck, Jessica G. Woo

**Affiliations:** 1 University of Cincinnati College of Medicine, Cincinnati, OH, USA; 2 Division of Biostatistics and Epidemiology, Cincinnati Children’s Hospital Medical Center, Cincinnati, OH, USA

**Keywords:** Dietary Approaches to Stop Hypertension diet, CVD, Micronutrients, Macronutrients, Nutrition, Epidemiology

## Abstract

Diet is a contributing factor to CVD risk, but how diet quality changes over the long term and contributes to CVD risk is less well studied. Diet data were analysed from parents and offspring from the Princeton Lipid Research Study (24-h recall in the 1970s; Block FFQ in 1998). Diet quality was assessed using an 8-point Dietary Approaches to Stop Hypertension nutrient-based scoring index, including a new method for scoring in children, as well as examining twelve key macro/micronutrients. Outcomes included blood glucose, blood pressure, serum lipids and BMI. The analysis included 221 parents (39 % male, mean age 38·9 ± 6·5 at baseline and 66·6 ± 6·6 at follow-up) and 606 offspring (45 % male, 11·9 ± 3·2 at baseline and 38·5 ± 3·6 at follow-up). Parents’ Dietary Approaches to Stop Hypertension score increased slightly from baseline to follow-up (1·4 ± 1·0 and 2·1 ± 1·3, respectively, *P* < 0·001), while offspring remained consistent (1·6 ± 0·9 and 1·6 ± 1·1, respectively, *P* = 0·58). Overall, the Dietary Approaches to Stop Hypertension score, adjusted for age, race, sex and BMI, was not significantly associated with any examined outcomes. Of the macro/micronutrients at follow-up, saturated and total fat were associated with increased diabetes and dyslipidaemia in parents, while the inverse was seen with niacin. Among offspring, niacin was associated with lower rates of hypertension and dyslipidaemia. In conclusion, no relationship was detected between Dietary Approaches to Stop Hypertension adherence and disease outcomes. However, both saturated fat and niacin were associated with components of CVD risk, highlighting the need for improved diet quality overall.

CVD is the leading cause of death in the USA totalling 697 000 deaths yearly, and related co-morbidities such as obesity, hypertension (HTN), diabetes and dyslipidaemia have been increasing in the past 20 years^(1)^. One factor contributing to high rates of heart disease is poor dietary intake in adults. Previous research has identified that diets high in sugar, Na and fat intake while low in fibre, fruits and vegetables contribute to the development of heart disease in adults^(2–4)^ and increase the risk for other cardiometabolic diseases that contribute to heart disease, including diabetes^(5)^, HTN^(6)^, obesity^(7)^ and dyslipidaemia^(8)^. This dietary pattern is extremely common in the USA, such that few adults achieve an ideal diet score, as recommended by the American Heart Association (≥4·5 cups/d of fruit and vegetables; ≥ 2 servings/week of fish; ≥3 servings/d of whole grains; <450 kcal/week of sugar-sweetened beverages; and <1500 mg/d of Na)^(9)^. Efforts to improve adults’ diets have resulted in the critical evaluation of several dietary plans, including the Dietary Approaches to Stop Hypertension (DASH) eating plan, which was developed as a potential therapy to reduce HTN. The DASH diet has been found in randomised controlled trials to result in significant reductions in blood pressure (BP)^(10)^, obesity^(11)^ and dyslipidaemia^(12)^. These findings have made the DASH diet one of the key lifestyle ‘prescriptions’ for reducing cardiovascular risk in adults.

Although the clinical manifestations of CVD may not be evident until adulthood, the developmental roots extend back into childhood when many health habits are established. Childhood diets low in fruit and vegetable intake and high in fat are related to worse cardiometabolic profiles in childhood^(13,14)^. However, little is known about how childhood dietary habits are maintained into adulthood or how child-to-adult changes in diet may influence disease risk. A study done as part of the Minnesota Heart Health Program tracked school-aged children from 1983 to 1989 and found evidence for early solidification and ‘tracking’, or preservation of relative position, of physical activity, food choices and smoking^(15,16)^. This is consistent with other studies reporting mild-to-moderate tracking of diet intake and quality along with additional lifestyle behaviours^(17,18)^.

This study adds to the limited research surrounding the relationship between childhood diet, adult diet and cardiovascular risk/disease. The objectives were to investigate the correlation between diet and cardiovascular risk factors in childhood and adulthood among both parents and children. In addition, we aimed to evaluate how these associations may change over the course of a 25–30-year follow-up. We hypothesised that diets lacking in key nutrients for ideal cardiovascular health would be conserved over time in both generations and be reflected as increased blood glucose, BP, serum lipids and BMI. The goal of determining the longitudinal implications of diet composition is to provide a window into effectively promoting ideal cardiovascular health.

## Methods

### Data source

Data were examined from the existing 1973–1978 National Institutes of Health-National Heart, Lung, and Blood Institute Lipid Research Clinics (LRC) Princeton Lipid Study and the 1998 Princeton Follow-up Study (PFS), collectively referred to as the Princeton Lipid Research Study. Both the LRC and the PFS have been extensively detailed elsewhere^(19–22)^. Briefly, during the three-visit series conducted in the 1970s, data were collected on schoolchildren (K-12) and a 50 % random sampling of parents in each household. An initial visit collected baseline serum lipid levels, demographic information and household composition. Approximately 6 weeks later, a random selection of participants along with all hyperlipidaemic individuals was recalled. Interviewers at Visit 2 (baseline) collected demographic, anthropometric and dietary data as well as laboratory measurements and BP. The PFS (follow-up) was a longitudinal reassessment of that cohort that included families from the LRC study in order to examine 25–30-year changes in CVD risk factors. Researchers collected updated health history and similar data as in the 1970s. For the present analysis, only parents and offspring with diet data at both baseline in the 1970s and follow-up between 1998 and 2003 (PFS) were included (online Supplementary Fig. 1). Clinical data from all visits were used as necessary. Comparisons of included and excluded participants are provided in online Supplementary Table 1.

This study was conducted according to the guidelines laid down in the Declaration of Helsinki, and all procedures involving human subjects/patients were approved by the Cincinnati Children’s Hospital Medical Center Institutional Review Board. For the original studies, adults (parents and offspring at the adult visit) provided written informed consent and offspring at baseline also provided assent if they were at least 11 years old. For this secondary analysis, the data from the Princeton Lipid Research Study Repository were used (IRB no. 2012-0257), and the Cincinnati Children’s Hospital Medical Center Institutional Review Board determined this was an exempt study (IRB no. 2015-9102).

### Measurements and outcomes

Data obtained from the LRC study included nutrient intake computed from a single 24-h dietary recall; data on BP, lipid profile, serum glucose and BMI where available; prescribed medications; and sex, age and race. The methods of measurement collection have been described previously^(19–22)^. Briefly, BP was taken from the right arm after sitting for 5 min, and the mean of two readings was used. Plasma cholesterol, TAG and lipoproteins were collected after ≥12 h of fasting, and anyone not fasting was excluded. Lipid levels were quantified following the methods of the LRC Laboratory Methods Manual^(20)^. Height and weight (measured in light indoor clothing with shoes removed) were used to calculate BMI in kg/m^2^. Study participants reported their own food intake, and the final data included only those recalls judged reliable by the interviewer. Nutrient data in the 24-h recall were centrally coded based on food products from the US Department of Agriculture Handbook No. 8^(23)^. Data from PFS were identical as above except where listed: nutrient intake came from the Block FFQ, and sex, age, race, marital status, annual household income and highest education level were obtained by self-report for the subject themself.

Outcome categories were based solely on values measured during the study, rather than on medication use or reported disease diagnosis. Childhood outcomes for the offspring in the 1970s were based on cutoffs for glucose intolerance, high BP, dyslipidaemia and obesity used clinically around the time of the study to more accurately depict disease classifications in the 1970s (online Supplemental Table 2). Glucose intolerance was defined as ≥6.11 mmol/l fasting glucose^(24)^; high BP cutoff was ≥90th percentile in systolic or diastolic blood pressure for age and sex^(25)^; dyslipidaemia was defined as TAG ≥1.69 mmol/l, or HDL-cholesterol ≤1.03 mmol/l in males, or HDL-cholesterol ≤ 1.29 mmol/l in females^(26)^; overweight/obesity was determined as BMI ≥ 85th percentile for age and sex using the Centers for Disease Control and Prevention growth charts^(27)^.

Adulthood conditions were categorised into groups of normal *v*. moderate to high based on accepted criteria at the time of the follow-up study in the early 2000s (online Supplementary Table 3). The following outcomes were evaluated in this study: diabetes^(28)^, HTN^(29,30)^, dyslipidaemia^(31)^ and obesity^(32,33)^. Participants were rated as moderate or high in the respective disease category if they met one cutoff value (diabetes and obesity) or one or more of the cutoff values where multiple existed (systolic blood pressure or diastolic blood pressure for HTN; LDL-cholesterol or TAG or HDL-cholesterol or total cholesterol (TC) for dyslipidaemia). They were rated as normal if they met all ‘normal’ criteria. For example, the outcome ‘diabetes’ was a combination of individuals who were ranked as moderate, or prediabetic, as well as high, or diabetic.

### DASH score

Unlike newer studies, our data did not include food groups or serving size information that would typically be used for DASH scoring, thus necessitating a nutrient-based scoring approach. To examine diet quality in adults, we used a nutrient-based DASH score previously generated by Mellen *et al.* ^(34)^. Macronutrients were converted to percentage of total energy, and micronutrients were converted to mg or g/1000 kcal (4184 kJ) to adjust for differences in individual energy consumption. DASH goals and intermediate targets identified by Mellen include nine nutrients (saturated fat, total fat, protein, cholesterol, fibre, Mg, Ca, K and Na). However, given the limitations in the baseline diet data, we did not include Mg and only counted fibre from grain sources in our final scoring. We modified the follow-up DASH score to be comparable. Individuals who met the goal for each component received 1 point, those who met an intermediate goal received 0·5 points and those who met neither goal received 0 points. Thus, our total possible DASH score was out of 8 rather than 9 points. Adherence to a modified DASH diet was defined as a DASH score of ≥ 4·0. This ‘modified DASH’ scoring was applied to the baseline 24-h recall data for parents and all the Block FFQ data.

DASH targets based on nutrients with intermediate scoring for children had not previously been generated. In order to score offspring baseline diet data in a manner consistent with adult scoring, we created a childhood DASH score based on previous work by Cohen^(35)^. Offspring were split into three age groups: 6–10·99, 11–13·99 and 14–19·99. Using the 2015–2020 dietary guidelines^(4,36)^, we determined the average energy intake for each group based on the moderately active category (physical activity equivalent to walking about 1·5–3 miles/d). Total kilocalories for age groups 6–10 and 11–13 were averaged across sex; however, average calories in the 14–19 group were sex-specific due to large discrepancies in energy needs by sex in this age group. We created an intermediate target group for each childhood age category in the new DASH score based on interpolation from the Mellen intermediate scores in adults (online Supplementary Table 4). Like in the adult scoring, we did not include Mg when scoring the offspring.

In addition, diet quality was assessed using each of the individual components from the DASH score as well as other key nutrients included in the dietary guidelines^(4)^. Macronutrients included SFA (grams (g)), total fat (g), protein (g) and carbohydrates (g). Micronutrients included cholesterol (milligrams (mg)), fibre (g), Ca (mg), K (mg), Na (mg), Na:K ratio, Fe (mg), niacin (mg) and vitamin C (mg). Due to a lack of data at baseline, unsaturated fatty acids and sucrose were not included in the analysis. The nutrients were converted to a percentage of energy (macronutrients) or mg or g/1000 kcal (4184 kJ; micronutrients) to account for differences in total intake by age and method of assessment^(37)^. *Z*-scores for macro- and micronutrients were then calculated within visit by age group (≤8·99, 9–11·99, 12–14·99, 15–17·99, 18–19·99, 20–49·99, 50–69·99, ≥70) and sex by subtracting off the group-specific mean and dividing by the group-specific sd to account for age and sex differences in nutrient distributions by method of assessment. All nutrient analyses were conducted using these *z*-scores.

### Statistical analysis

All statistical analysis was done using Statistical Analysis Systems software version 9·4 (SAS Institute). Changes in DASH scores were assessed using paired *t* tests. Tracking of DASH score and specific nutrients by generation across time were examined using Spearman correlations. Logistic regression was used to examine the relationships between DASH diet score and specific nutrients with categorical outcomes. For continuous measurement outcomes of fasting plasma glucose, BP, lipids and BMI or BMI percentile, we conducted adjusted general linear regression. Covariates of interest, including age, race, sex, BMI or BMI percentile in offspring at baseline, where obesity was not the outcome examined, marital status (PFS), annual household income (PFS) and highest education level obtained by the subject (PFS), were tested and retained in the models if significant (*P* < 0·05).

## Results

The analysis included 221 parents (39 % male, mean age 38·9 ± 6·5 at baseline and 66·6 ± 6·6 years at follow-up) and 606 offspring (45 % male, mean age 11·9 ± 3·2 at baseline and 38·5 ± 3·6 years at follow-up). Parents were 80 % white, while offspring were 70 % white. The age distribution for offspring at baseline was 36 % aged 6–10 years, 29 % aged 11–13 years and 35 % aged 14–19 years (Table 1).

**Table 1.** Characteristics of the study population and disease outcomes

	Parents				Offspring			
	*n*		%		*n*		%	
Total *n* 827	221				606			
Sex, *n* (%)								
Male	86		39 %		271		45 %	
Female	135		61 %		335		55 %	
Race, *n* (%)								
White	176		80 %		427		70 %	
Other	45		20 %		179		30 %	
Offspring age group, years, *n* (%)								
6–10·99	–				215		36 %	
11–13·99	–				178		29 %	
14–19·99	–				213		35 %	
Marital status at PFS								
Married, *n* (%)	158		76 %		379		66 %	
Education level at PFS								
High school graduate or less	107		49 %		154		26 %	
Some college	68		31 %		201		33 %	
College graduate	32		15 %		165		27 %	
Graduate degree	12		6 %		82		14 %	
Annual household income (year 2000 dollars) at PFS								
<$10k	10		7 %		4		2 %	
$10–<$25k	41		28 %		20		9 %	
$25–<$50k	54		36 %		59		26 %	
$50–<$75k	23		15 %		53		24 %	
$75k or more	21		14 %		87		39 %	
	Baseline		Follow-up		Baseline		Follow-up	
	Mean or *n*	sd or %	Mean or *n*	sd or %	Mean or *n*	sd or %	Mean or *n*	sd or %
Age, years, mean (sd)	38·9	6·5	66·6	6·6	11·9	3·2	38·5	3·6
BMI, kg/m^2^, mean (sd)	26·0	4·6	30·0	6·1	19·8	4·4	28·6	7·0
DASH diet score, mean (sd)	1·4	1·0	2·1	1·3	1·6	0·9	1·6	1·1
Outcomes, *n* (%)								
Diabetes	7	3 %	54	24 %	4	1 %	37	6 %
* Medication, n (%)* *	2	29 %	26	48 %	–		11	30 %
Hypertension	53	24 %	98	44 %	70	12 %	120	20 %
* Medication, n (%)*	18	34 %	66	67 %	1	1 %	31	26 %
Dyslipidaemia†	181	82 %	183	83 %	212	35 %	458	75 %
* Medication, n (%)*	5	3 %	78	43 %	–		24	5 %
Obesity/overweight	37	17 %	175	79 %	61	10 %	393	65 %

PFS, Princeton Follow-up Study; DASH, Dietary Approaches to Stop Hypertension.

* Number of medications taken as a percentage of total individuals in disease outcome group; data on medication use for overweight/obesity were not collected.

† The number of dyslipidaemic individuals was largely influenced by HDL-cholesterol, which accounted for 30–40 % of individuals at baseline (43 % of dyslipidaemia in parents, 33 % of the offspring) and over 50 % of the values at follow-up (57 % parents, 56 % offspring).

In comparing those participants who were included *v*. excluded from the final analysis, there was no difference in sex, race, education level or income. Included offspring were somewhat younger than those excluded, and cholesterol and TAG were higher in both parents and offspring in the included group (online Supplementary Table 1).

### DASH scores

**Fig. 1.**
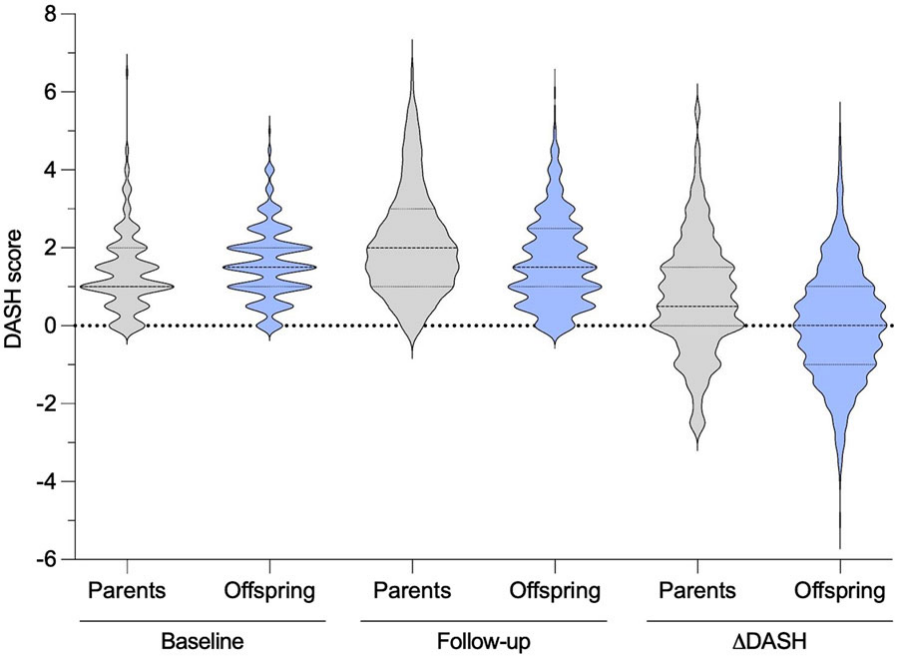
Change in the Dietary Approaches to Stop Hypertension (DASH) score in parents and offspring across 20–25 years. Discrete modified DASH scores at baseline (1970s) and follow-up (1998–2003) and change in DASH score for parents (grey) and offspring (blue). Mean and se represented by the darker and lighter dashed lines, respectively. Values in Fig. 1 appear discrete due to the half-point intervals on the DASH score.

The average total DASH score for the Block FFQ before (2·2 ± 1·5) and after modification (1·8 ± 1·2) were found to be highly correlated (0·97). Examination of the Bland–Altman plot for the difference in DASH score (follow-up minus baseline) within each generation (not shown) displayed little consistency in a score; however, there was some evidence of regression to the mean. Using the modified DASH score, parents’ total DASH score increased from baseline to follow-up (mean ± sd: 1·4 ± 1·0 and 2·1 ± 1·3, respectively, *P* < 0·001), while offspring diet quality remained consistent (1·6 ± 0·9 and 1·6 ± 1·1, respectively, *P* = 0·58; Fig. 1). Parents increased from 2·7 % adherent at baseline to 12·7 % adherent at follow-up (*P* = 0·005 for increase). Offspring increased from 3·1 % adherent at baseline to 5·3 % adherent at follow-up (*P* = 0·30).

**Fig. 2.**
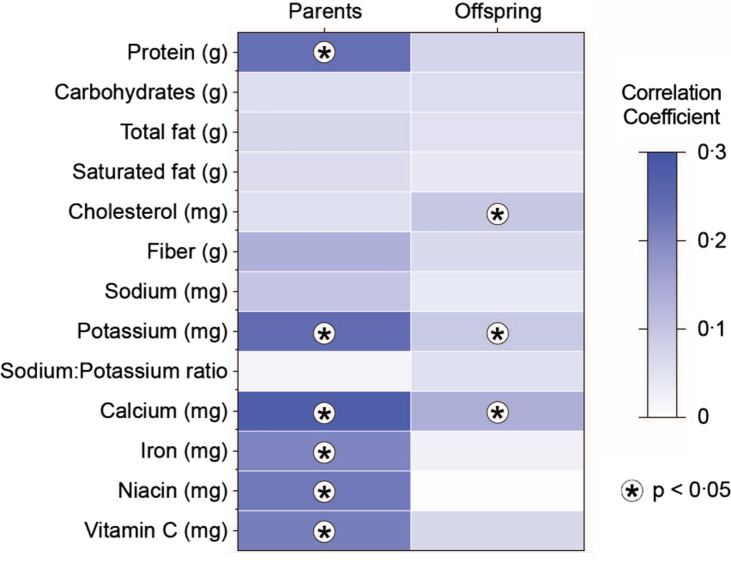
Specific nutrient tracking across 20–25 years, by generation. Spearman correlations between baseline and follow-up for parents and offspring. * indicates *P*-values <0·05 for difference from 0.

Macro- and micronutrient intake was also evaluated across visits using z-scores of the energy-adjusted nutrient values. Correlations across time showed small, yet significant correlations in both parents and offspring (Fig. 2). Significant correlations in the parents were protein, K, Ca, Fe, niacin and vitamin C. Significant nutrients in the offspring were cholesterol, K and Ca. Overall correlations were weak, with none of the values in parents surpassing 0·3 and none of the values in the offspring surpassing 0·15.

### Relation to outcomes

Table 2 presents adjusted OR and 95 % CI for concurrent (same visit) and longitudinal (across visit) relationships of the DASH score with disease outcomes, adjusting for sex, race, age and BMI at the visit, except for models of obesity. None of the adjusted OR for concurrent relationships at either baseline or follow-up for either generation was significant. Similarly, relationships of baseline DASH score with follow-up outcomes were NS for either generation. In evaluating the change in DASH score (follow-up minus baseline), a greater increase in DASH score was associated with lower rates of dyslipidaemia (*P* = 0·04) among the offspring.

**Table 2.** Adjusted odds ratios and confidence intervals for Dietary Approaches to Stop Hypertension (DASH) diet score *v*. disease outcomes, by generation

	Baseline outcomes							
	Diabetes		Hypertension		Dyslipidaemia		Obesity	
	OR	95 % CI	OR	95 % CI	OR	95 % CI	OR	95 % CI
Parents								
Baseline DASH*	0·78	0·30, 2·04	1·13	0·78, 1·64	0·98	0·74, 1·31	1·54	0·99, 2·05
	Glucose intolerance		High blood pressure		Dyslipidaemia		Obesity	
Offspring								
Baseline DASH*	0·27	0·06, 1·32	1·08	0·86, 1·36	0·96	0·79, 1·18	1·31	0·99, 1·73
	Follow-up outcomes							
	Diabetes		Hypertension		Dyslipidaemia		Obesity	
Parents								
Baseline DASH score*	0·82	0·57, 1·19	1·17	0·86, 1·61	0·80	0·54, 1·19	1·07	0·74, 1·54
Follow-up DASH score†	0·86	0·66, 1·11	0·86	0·69, 1·07	0·81	0·62, 1·06	1·04	0·81, 1·34
Change in DASH (follow-up minus baseline)*	0·94	0·75, 1·19	0·90	0·73, 1·10	0·93	0·72, 1·21	1·01	0·80, 1·28
Offspring								
Baseline DASH score*	0·93	0·62, 1·37	1·13	0·88, 1·44	1·25	1·00, 1·55	1·03	0·85, 1·24
Follow-up DASH score†	0·97	0·68, 1·28	0·93	0·78, 1·11	0·94	0·78, 1·13	0·94	0·80, 1·09
Change in DASH (follow-up minus baseline)*	0·98	0·76, 1·27	0·89	0·76, 1·05	0·86	0·75, 0·99	0·95	0·84, 1·07

* Baseline Dietary Approaches to Stop Hypertension (DASH) and change in DASH score models adjusted by sex, race, age at baseline and BMI at baseline (except obesity).

† Follow-up DASH adjusted by sex, race, age at Princeton Follow-up Study (PFS) and BMI at PFS (except obesity).

Bolded value indicates *P* = 0.04.

We also examined diet using 12 macro/micronutrients and their relation to continuous risk factor measurements using adjusted linear regression at baseline (Table 3) and follow-up (Table 4), by generation. Several associations were statistically significant although overall values were small. Cholesterol, Na, Na:K ratio, Ca, Fe and vitamin C were all associated with three or fewer continuous variables and zero to one categorical disease outcomes. At baseline among parents, total fat had a negative association with lipid levels, while K and niacin intake appeared beneficial (higher fat was associated with higher LDL-cholesterol, higher K and niacin with higher HDL-cholesterol). In addition, higher fibre was seen with higher glucose, and higher protein was associated with higher BMI. In offspring at baseline, carbohydrates, total fat and saturated fat were all associated with TAG, although not all with consistent directionality (increased carbohydrate intake was seen with a small increase in TAG, while an increase in total fat and saturated fat was associated with a small decrease). Higher protein and fibre were associated with higher LDL-cholesterol and TC, respectively.

**Table 3.** Regression coefficients and standard error for specific nutrients and disease outcomes at baseline, by generation

Nutrient (Z-score)^b^	Glucose (mmol/L)	SBP (mmHg)	TC (mmol/L)	LDL-C (mmol/L)	HDL-C (mmol/L)	TG^a^ (mmol/L)	BMI (kg/m^2^)
**Parents**							
Protein	−0·05 ± 0·06	0·04 ± 0·85	0·02 ± 0·07	0·01 ± 0·06	0·03 ± 0·02	−0·001 ± 0·0003	**0·69 ± 0·30**
Carbohydrates	−0·03 ± 0·07	−0·69 ± 0·92	−0·15 ± 0·08	−0·13 ± 0·07	−0·05 ± 0·02	0·00 ± 0·0005	−0·44 ± 0·33
Total fat	0·04 ± 0·08	−1·77 ± 1·0	0·11 ± 0·09	**0·15 ± 0·08**	−0·001 ± 0·03	0·0003 ± 0·0004	−0·30 ± 0·36
Saturated fat	0·08 ± 0·07	−0·65 ± 0·97	0·12 ± 0·08	0·12 ± 0·07	0·01 ± 0·02	0·0003 ± 0·0004	−0·15 ± 0·35
Cholesterol	−0·02 ± 0·07	1·14 ± 0·89	0·04 ± 0·08	0·03 ± 0·07	0·03 ± 0·02	0·00 ± 0·0005	0·25 ± 0·32
Fiber	**0·14 ± 0·06**	−1·16 ± 0·83	−0·10 ± 0·07	−0·06 ± 0·06	0·01 ± 0·02	−0·001 ± 0·0003	−0·9 ± 0·30
Sodium	0·04 ± 0·07	1·20 ± 0·86	−0·07 ± 0·07	−0·07 ± 0·07	**0·03 ± 0·02**	−0·001 ± 0·0003	−0·35 ± 0·31
Potassium	0·08 ± 0·07	0·97 ± 0·86	−0·12 ± 0·07	−0·11 ± 0·07	**0·05 ± 0·02**	−0·001 ± 0·0005	−0·08 ± 0·31
Na:K ratio	0·01 ± 0·11	0·30 ± 1·41	0·04 ± 0·12	0·05 ± 0·11	0·01 ± 0·04	0·0001 ± 0·001	−0·40 ± 0·51
Calcium	0·05 ± 0·06	0·98 ± 0·81	−0·05 ± 0·07	−0·07 ± 0·06	0·04 ± 0·02	0·0002 ± 0·0003	0·06 ± 0·29
Iron	−0·06 ± 0·07	1·69 ± 0·92	−0·10 ± 0·08	−0·09 ± 0·07	**0·06 ± 0·02**	−0·001 ± 0·0005	0·27 ± 0·33
Niacin	−0·09 ± 0·07	1·39 ± 0·95	−0·03 ± 0·08	−0·02 ± 0·07	**0·05 ± 0·02**	−0·0006 ± 0·0005	0·41 ± 0·34
Vitamin C	**0·21 ± 0·07**	−0·02 ± 0·92	−0·11 ± 0·08	−0·09 ± 0·07	0·01 ± 0·02	0·0001 ± 0·0005	0·44 ± 0·33
**Offspring**							
Protein	−0·02 ± 0·02	0·20 ± 0·44	0·07 ± 0·04	**0·07 ± 0·03**	0·01 ± 0·01	−0·0002 ± 0·0002	0·29 ± 0·16
Carbohydrates	−0·01 ± 0·02	0·34 ± 0·44	−0·03 ± 0·19	−0·03 ± 0·03	−0·01 ± 0·01	**0·0006 ± 0·0002**	0·02 ± 0·16
Total fat	0·01 ± 0·02	−0·47 ± 0·43	0·002 ± 0·04	0·001 ± 0·03	0·01 ± 0·01	**−0·0006 ± 0·0002**	−0·13 ± 0·16
Saturated fat	0·02 ± 0·02	−0·53 ± 0·44	0·007 ± 0·04	0·02 ± 0·03	0·01 ± 0·01	**−0·0005 ± 0·0002**	−0·21 ± 0·16
Cholesterol	−0·003 ± 0·02	−0·36 ± 0·42	−0·03 ± 0·04	−0·04 ± 0·03	0·003 ± 0·01	−0·0001 ± 0·0002	0·11 ± 0·16
Fiber	−0·03 ± 0·02	−0·32 ± 0·40	**0·07 ± 0·03**	0·04 ± 0·03	0·01 ± 0·01	0·0002 ± 0·0002	−0·06 ± 0·15
Sodium	0·02 ± 0·02	−0·31 ± 0·43	−0·001 ± 0·04	0·0003 ± 0·03	−0·002 ± 0·01	0·0001 ± 0·0002	−0·05 ± 0·16
Potassium	0·02 ± 0·02	−0·39 ± 0·44	−0·008 ± 0·04	−0·003 ± 0·03	−0·01 ± 0·01	−0·0001 ± 0·0002	−0·22 ± 0·16
Na:K ratio	−0·01 ± 0·03	0·46 ± 0·61	−0·03 ± 0·05	−0·03 ± 0·05	−0·01 ± 0·02	0·0003 ± 0·0003	0·07 ± 0·22
Calcium	0·02 ± 0·02	−0·11 ± 0·47	−0·002 ± 0·04	0·004 ± 0·04	0·01 ± 0·01	−0·0003 ± 0·0002	**−0·40 ± 0·17**
Iron	0·004 ± 0·02	−0·15 ± 0·42	0·002 ± 0·04	0·01 ± 0·03	−0·001 ± 0·01	0·0002 ± 0·0002	−0·08 ± 0·15
Niacin	0·01 ± 0·02	0·04 ± 0·42	−0·01 ± 0·04	−0·01 ± 0·03	−0·004 ± 0·01	0·0001 ± 0·0002	0·01 ± 0·16
Vitamin C	−0·02 ± 0·02	−0·07 ± 0·40	−0·04 ± 0·03	−0·04 ± 0·03	−0·002 ± 0·01	0·0002 ± 0·0002	−0·14 ± 0·15

Regression coefficient ± standard error of the mean from adjusted linear regression analysis for continuous disease outcomes at baseline for parents and offspring. Bolded coefficients represent values for which *p* < 0.05. Adjustments were made for sex, race, age, and body mass index where BMI was not the outcome measured.

TC, total cholesterol (mmol/L); LDL-C, low density lipoprotein cholesterol (mmol/L); HDL-C, high density lipoprotein cholesterol (mmol/L); TG, triglycerides (mmol/L); SBP, systolic blood pressure (mmHg); BMI, body mass index (kg/m^2^); Na:K ratio, sodium to potassium ratio.

a Triglycerides were log transformed to account for skew.

b Age and sex-specific *z*-scores of nutrient intake, adjusted for total energy intake.

**Table 4.** Regression coefficients and standard error for specific nutrients and disease outcomes at follow-up, by generation

Nutrient (Z-score)^b^	Glucose (mmol/L)	SBP (mmHg)	TC (mmol/L)	LDL-C (mmol/L)	HDL-C (mmol/L)	TG^a^ (mmol/L)	BMI (kg/m^2^)
**Parents**							
Protein	−0·02 ± 0·16	2·02 ± 1·34	0·01 ± 0·07	0·03 ± 0·07	0·03 ± 0·02	−0·0003 ± 0·005	−0·01 ± 0·43
Carbohydrates	−0·22 ± 0·16	−1·62 ± 1·28	**−0·21 ± 0·07**	**−0·16 ± 0·06**	**−0·08 ± 0·02**	0·0007 ± 0·0005	−0·53 ± 0·41
Total fat	**0·33 ± 0·16**	1·80 ± 1·34	**0·24 ± 0·07**	**0·21 ± 0·07**	0·05 ± 0·02	−0·0005 ± 0·0005	**0·83 ± 0·42**
Saturated fat	**0·40 ± 0·17**	0·74 ± 1·38	**0·20 ± 0·07**	**0·19 ± 0·07**	0·03 ± 0·02	−0·0005 ± 0·0005	0·81 ± 0·43
Cholesterol	0·22 ± 0·16	1·28 ± 1·37	0·08 ± 0·08	0·05 ± 0·07	**0·07 ± 0·02**	−0·0005 ± 0·0005	**0·95 ± 0·43**
Fiber	−0·13 ± 0·16	−2·14 ± 1·33	−0·13 ± 0·07	−0·09 ± 0·07	−0·03 ± 0·02	0·0002 ± 0·0005	−0·60 ± 0·42
Sodium	0·17 ± 0·18	−0·07 ± 1·49	0·10 ± 0·08	0·09 ± 0·08	−0·02 ± 0·03	0·0005 ± 0·0005	0·22 ± 0·48
Potassium	−0·07 ± 0·17	−0·67 ± 1·39	**−0·23 ± 0·08**	**−0·16 ± 0·07**	−0·03 ± 0·02	0·00 ± 0·0005	−0·28 ± 0·44
Na:K ratio	0·20 ± 0·16	0·30 ± 1·41	**0·25 ± 0·07**	**0·15 ± 0·07**	0·01 ± 0·02	0·0005 ± 0·0005	0·32 ± 0·43
Calcium	−0·05 ± 0·16	−0·09 ± 1·36	−0·11 ± 0·07	−0·05 ± 0·07	−0·02 ± 0·02	0·0001 ± 0·0005	−0·46 ± 0·43
Iron	−0·24 ± 0·15	−0·57 ± 1·21	−0·06 ± 0·07	−0·04 ± 0·06	−0·03 ± 0·02	0·0003 ± 0·0003	−0·64 ± 0·38
Niacin	**−0·39 ± 0·16**	0·97 ± 1·34	−0·05 ± 0·07	−0·03 ± 0·07	0·03 ± 0·02	−0·0003 ± 0·0005	**−0·92 ± 0·42**
Vitamin C	0·07 ± 0·16	−1·34 ± 1·28	**−0·26 ± 0·07**	−0·21 ± 0·06	−0·04 ± 0·02	0·00 ± 0·0003	−0·01 ± 0·41
**Offspring**							
Protein	**0·19 ± 0·07**	−1·12 ± 0·58	0·003 ± 0·05	−0·04 ± 0·04	**0·04 ± 0·01**	−0·0002 ± 0·0002	0·33 ± 0·30
Carbohydrates	−0·04 ± 0·07	−0·94 ± 0·57	−0·05 ± 0·05	0·0005 ± 0·04	**−0·05 ± 0·01**	0·0001 ± 0·0002	−0·08 ± 0·29
Total fat	0·03 ± 0·07	0·73 ± 0·57	0·07 ± 0·05	0·06 ± 0·04	0·01 ± 0·01	−0·0001 ± 0·0002	0·27 ± 0·29
Saturated fat	−0·06 ± 0·07	0·86 ± 0·60	0·05 ± 0·05	0·03 ± 0·04	0·01 ± 0·01	−0·0001 ± 0·0002	0·26 ± 0·29
Cholesterol	−0·01 ± 0·08	−0·03 ± 0·61	0·02 ± 0·05	−0·03 ± 0·04	**0·04 ± 0·02**	−0·0001 ± 0·0002	**0·95 ± 0·30**
Fiber	0·11 ± 0·07	**−1·55 ± 0·56**	−0·06 ± 0·04	−0·04 ± 0·04	**0·04 ± 0·01**	**−0·0008 ± 0·0002**	−0·39 ± 0·29
Sodium	0·06 ± 0·07	−0·45 ± 0·57	0·05 ± 0·05	0·0008 ± 0·04	**0·04 ± 0·01**	−0·0001 ± 0·0002	−0·19 ± 0·29
Potassium	0·03 ± 0·07	**−1·69 ± 0·58**	−0·04 ± 0·05	−0·05 ± 0·04	0·03 ± 0·01	−0·0005 ± 0·0002	−0·45 ± 0·30
Na:K ratio	−0·01 ± 0·07	**1·34 ± 0·57**	0·07 ± 0·05	0·04 ± 0·04	0·003 ± 0·01	0·0005 ± 0·0002	0·20 ± 0·29
Calcium	0·12 ± 0·07	−0·38 ± 0·59	−0·02 ± 0·05	−0·05 ± 0·04	0·01 ± 0·01	0·0001 ± 0·0002	−0·29 ± 0·30
Iron	0·07 ± 0·08	**−2·45 ± 0·58**	−0·02 ± 0·05	−0·01 ± 0·04	0·004 ± 0·02	−0·0002 ± 0·0002	−0·42 ± 0·30
Niacin	0·12 ± 0·07	**−2·10 ± 0·56**	−0·0 ± 0·05	−0·01 ± 0·04	**0·03 ± 0·01**	**−0·0006 ± 0·0002**	−0·18 ± 0·29
Vitamin C	−0·06 ± 0·07	−0·63 ± 0·58	−0·08 ± 0·05	−0·07 ± 0·04	0·004 ± 0·01	−0·0001 ± 0·0002	0·42 ± 0·30

Regression coefficient ± standard error of the mean from adjusted linear regression analysis for continuous disease outcomes at follow-up for parents and offspring. Bolded coefficients represent values for which *p* < 0.05. Adjustments were made for sex, race, age, and body mass index where BMI was not the outcome measured.

TC, total cholesterol (mmol/L); LDL-C, low density lipoprotein cholesterol (mmol/L); HDL-C, high density lipoprotein cholesterol (mmol/L); TG, triglycerides (mmol/L); SBP, systolic blood pressure (mmHg); BMI, body mass index (kg/m^2^); Na:K ratio, sodium to potassium ratio.

a Triglycerides were log transformed to account for skew.

b Age and sex-specific *z*-scores of nutrient intake, adjusted for total energy intake.

At follow-up among parents, higher total fat and saturated fat intake was associated with higher glucose measurements, while higher niacin was associated with lower glucose. Increased intake of carbohydrates and K was negatively associated with lipids (higher carbohydrates with lower TC, LDL-cholesterol, HDL-cholesterol, higher K with lower TC, LDL-cholesterol), while increased amounts of total fat and saturated fat were positively associated with increased TC and LDL-cholesterol. A greater intake of total fat was also associated with a higher BMI, while a greater intake of niacin was seen with a lower BMI. Among offspring at follow-up, higher protein and niacin intake was associated with higher HDL-cholesterol, while the inverse was true of carbohydrates. Lower fibre and niacin were associated with higher TAG and systolic blood pressure. Higher systolic blood pressure was also seen with lower K intake, and higher glucose values were seen with increased intake of protein.

We then conducted adjusted logistic regression with categorical disease outcomes of diabetes, HTN, dyslipidaemia and obesity (online Supplementary Tables 5 and 6). Of the significant associations in the continuous outcomes, the following associations were also seen in the categorical outcomes. Among parents at baseline, the percentage of kcals contributed by protein, total fat and K increased the odds of obesity, diabetes and dyslipidaemia, respectively. In the follow-up parental generation, increased total fat and saturated fat and decreased niacin were associated with increased rates of diabetes. Among offspring at follow-up, increased protein intake was associated with higher diabetes rates, while lower K and niacin intake were associated with higher rates of HTN. Lower fibre, K and niacin intake were seen with higher odds of dyslipidaemia.

## Discussion

Our research adds a longitudinal context from childhood to adulthood to the conversations surrounding the impacts of lifespan changes in diet in large populations. The study of two generations followed for about 25 years showed that diet quality, as measured by DASH, was very low, and changes over time were small. While both generations improved in adherence to the DASH score, this increase was larger in the parents, and overall percentages remained low. Modification of the DASH scoring index with intermediate nutrient targets for children has not been previously documented to our knowledge. This presents a new method useful for maintaining consistency in DASH scoring between children and adults, where intermediate DASH thresholds allow for capturing small improvements in diet that may not reach the full cutoff criteria.

This study is consistent with previous work in adults showing overall low DASH adherence. Since the advent of the DASH diet and its support by programmes like the National Institutes of Health’s High Blood Pressure Health Education Initiative, it has been one of the leading lifestyle changes promoted for individuals with HTN. However, even among individuals with HTN who likely benefit most from this diet, recent studies have shown poor adherence to DASH and recommended further efforts to support the adoption of this diet by the public^(34)^. Data from the National Health and Nutrition Examination Survey (NHANES) have found that not only do adults in the USA have a poor adherence to the DASH diet, but adherence even deteriorated from 1994 to 2004^(34)^. More recent examination of NHANES data from 2001 to 2010 found a median total DASH score of 3·0 (of 9), with 78·3 % scoring < 4·5, which was the threshold for adherence^(38)^. While the DASH diet was designed for adults, one study used NHANES data to apply DASH scoring to elementary through high school students and similarly found very poor adherence^(35)^. The introduction of the DASH diet in 1997 has been counteracted by nationwide trends of increasing fast-food and sweetened beverage intake, as well as larger portion sizes^(39)^.

Dietary tracking of micro/macronutrient intake over time was low, with somewhat greater tracking seen within the parental generation. Previous studies have suggested mild-to-moderate tracking of dietary intake from childhood/adolescence into adulthood, with particularly low tracking of the ‘western diet’^(40–43)^. Our findings suggest that certain nutrients in mid-adulthood track into later adulthood, while the tracking from childhood to adulthood is much weaker. Consistent with our findings, a recent study of 4733 children found diet only weakly tracked from early to late childhood^(44)^. Together, these findings could suggest that diet becomes more fixed over an individual’s life, with key transition periods being childhood into adolescence and adolescence into adulthood. With the hypothesis that childhood diet is more malleable, childhood may be a crucial window for intervention.

While the benefits of DASH have been proven in many intervention studies^(6,12,45)^, we were unable to identify those effects, likely given low adherence to this diet in a free-living population. Contrary to our hypothesis, we found no relationship between the DASH diet score and concurrent risk factors for CVD at baseline or follow-up. In a study applying DASH scoring to children and adolescents, a small inverse association between DASH score and systolic BP was noted; however. no other CVD risk factors were associated^(35)^. Larger epidemiological cohorts with individuals achieving higher DASH scores would be needed to determine the effects of full DASH adherence with cardiometabolic disease risk in a free-living population. One feature of the DASH diet is the synergy between nutrients that the entirety of the diet provides. Unfortunately, with so few participants reaching even intermediate scores for each component, this synergy is likely lost. We propose that there may be a threshold effect to the DASH diet and that small variations within low DASH scores have little overall impact on CVD.

While the DASH score was minimally associated with disease outcomes in this study, three specific nutrients were most frequently correlated to concurrent continuous and categorical disease outcomes. Of those nutrients tested, higher total fat and saturated fat intake were associated with significantly higher blood glucose, TC and LDL-cholesterol measurements in parents at follow-up, as well as higher odds of diabetes. Higher total fat intake was also associated with higher rates of diabetes in parents at baseline. SFA consumption is inconsistently associated with insulin resistance and diabetes in the literature. Some short-term randomised control trials have shown that saturated fat consumption may worsen insulin sensitivity and increase plasma glucose; however, other long-term trials and meta-analyses have found no association^(46–50)^. Recommendations from the AHA surrounding dietary fats and CVD outcomes date back to the 1960s^(51)^. Saturated fat in particular is well known to raise LDL-cholesterol and contribute to atherosclerosis^(52)^, and our data are consistent with prior findings linking saturated fat to CVD and CVD-associated mortality^(53,54)^.

Current recommendations to improve cardiovascular health focus on improving overall diet quality by replacing SFA with PUFA and increasing consumption of fish (a key source of PUFA), rather than solely reducing saturated fat or total fat intake^(51,55)^. In particular, dietary intake or supplementation of the conditionally essential *n*-3 PUFA EPA and DHA have been shown in some, but not all, randomised trials and meta-analyses to reduce CVD events, especially at higher doses in higher-risk patients^(56,57)^. These PUFA appear to act through several mechanisms including BP reductions, improvements in endothelial function and TAG-lowering effects^(58)^. Unfortunately, the present study could not assess PUFA intake directly but is broadly consistent with these other findings.

Niacin intake among offspring at follow-up was associated with decreased rates of HTN and lower systolic blood pressure as well as lower rates of dyslipidaemia, lower TAG and higher HDL-cholesterol. Niacin intake in parents at follow-up was also associated with lower rates of diabetes and lower blood glucose. Niacin has long been known to play a role in serum lipid profiles, through actions related to both lowering TAG and increasing HDL-cholesterol ^(59)^, and the results of this study suggest niacin’s impact on CVD health extends beyond lipids alone. Niacin supplementation has been shown in a meta-analysis of many randomised control trials to increase HDL-cholesterol, and similar findings have been documented with dietary-based niacin intake in free-living populations^(60,61)^. There is considerably less research on dietary intake of niacin and its impact on BP; however, some studies have suggested a BP-lowering effect when taking niacin supplements in the long term^(62)^. Finally, our findings linking niacin intake to diabetes appear consistent with limited previous literature. One study conducted with NHANES data found similar results and also suggested a threshold for the protective effect of niacin at 15·01 mg/d^(63)^. A recent meta-analysis of randomised control trials linked niacin supplementation to lower blood glucose^(64)^. Importantly, however, a systematic review of niacin randomised trials found no evidence for niacin in the prevention of CVD events or deaths^(65)^. Although these findings suggest several key associations between nutrients and risk factors for CVD, evaluation of individual nutrients does not provide the full picture of dietary quality, and micronutrient-specific analyses may be overlooking interactions among dietary components.

One important limitation to this study was the use of two different dietary survey methods. The single 24-h recall method is widely used due to its simplicity and convenience, including for all NHANES data gathered before 2002; however, it only provides a snapshot of an individual’s dietary intake and inherently lacks variation. Generally, the 24-h recall under-represents an individual’s true energy intake^(66,67)^. Alternatively, FFQ tend to overestimate food consumption and rely more on an accurate memory of overall items consumed^(68,69)^. Extensive validation of the Block questionnaire has been described elsewhere^(70,71)^. Another limitation to the FFQ is that by asking about specific food groups, it tends to be less inclusive of ethnic food choices if not designed to include heterogeneous diet choices^(72)^. Both types of data are self-reported and subject to participant bias. Unlike more recent diet studies that focus on foods rather than nutrients, only nutrient data were available for our study. Nutrient-based DASH scoring was used as a measure of diet quality to overcome the lack of data on specific foods. However, with no reported Mg or fibre from sources other than grains in the 24-h recall, this necessitated a modified DASH score. To limit the impact of different dietary assessment methods, we calculated within-visit energy-adjusted age- and sex-specific *z*-scores for each nutrient evaluated to limit distributional and mean differences by age, sex and visit. While the types of diet data collected in each study were somewhat different, we used Spearman correlations to test relative position within the distribution, rather than measured values.

In evaluating the differences between characteristics of those participants included *v*. excluded from the final analysis, there were several significantly higher lipid values in the included group. This could be related to the study enrolment method, which recalled both a random selection and participants with higher lipid values for all subsequent visits. Comparison of the randomly selected *v*. high lipid recalled groups at baseline largely accounted for these differences (data not shown). Additionally, in categorising individuals as ‘disease *v*. no disease’, our basis was limited to measurements that were later grouped into clinically relevant outcome categories. Unfortunately, we did not have key medication data (e.g. dose, duration, medication class) to be able to sufficiently account and adjust for medication usage in the overall analysis. The analysis thus classifies individuals whose measurements normalised after starting medication as not having a given diagnosis, representing a conservative approach to this analysis.

The lack of meaningful change in DASH score over time suggests that patients are not improving their diet after being diagnosed with HTN, dyslipidaemia or diabetes. This is contrary to a small body of literature describing positive dietary changes after a breast cancer diagnosis among European women^(73–75)^. This acts as a limitation to our study, as we were unable to determine the timeframe of dietary changes relative to disease diagnosis. We suggest that the minimal variability present in the low diet quality observed was not enough to detect any significant impact on disease, although it does not negate the possibility. Finally, further study is needed to validate the adapted nutrient-based DASH with intermediate scoring for children.

In conclusion, this study shows that specific nutrients are associated with cardiometabolic disease, including total and saturated fat with diabetes and dyslipidaemia, and niacin inversely with diabetes, HTN and dyslipidaemia. However, no significant associations between DASH diet quality scores and CVD outcomes were identified, likely due to low adherence to the DASH diet in this free-living population. Dietary tracking was seen most significantly in adulthood, suggesting greater malleability in early diet. Given that dietary changes are difficult to achieve and uncommon even after diagnosis of nutritionally related diseases, further efforts to establish and maintain high diet quality are needed.

## Supporting information

Beck and Woo supplementary material Beck and Woo supplementary material

## Data Availability

Data for the LRC Prevalence Study, comprising the first three visits in the 1970s, are available from the National Heart, Lung and Blood Institute BioLINCC resource (www.biolincc.nhlbi.nih.gov/studies/lrcps). Data from the follow-up study (1998–2003) can be obtained by request from Dr Woo.

## Supplementary material

For supplementary material/s referred to in this article, please visit https://doi.org/10.1017/S0007114524001521
