# Supplementary material for: The contribution of dietary composition over 25 years to cardiovascular risk factors in childhood and adulthood: the Princeton Lipid Research Study
Source: Br J Nutr. 2024 Oct 9;132(5):678–89. doi: 10.1017/S0007114524001521 (PMC11531936; doi:10.1017/S0007114524001521)
Supplement: Beck and Woo supplementary material [file S0007114524001521sup001.docx]

**Supplemental Figure 1: Flow diagram of Analysis Sample**

2652 seen at Visit 2 (854 parents and 1798 offspring)

1448 Seen at PFS (426 parents and 1022 offspring)

**Analysis Sample**

827 Seen at both Visit 2 and PFS with diet data (221 parents and 606 offspring)

593 not previously seen at Visit 2 (201 parents and 392 offspring)

1420 with PFS diet data (422 parents and 998 offspring)

2639 with Visit 2 diet data (848 parents and 1791 offspring)

1866 not eligible for PFS, not seen at PFS, or missing PFS diet data (627 parents and 1185 offspring)

**Supplemental Table 1: Comparison of PFS participants included vs. excluded from analysis**

|  | **Parents** | | | **Offspring** | | |  |
| --- | --- | --- | --- | --- | --- | --- | --- |
|  | **Included** | **Excluded** | **p-value** | **Included** | **Excluded** | **p-value** |  |
|  | 221 (52%) | 201 (48%) | -- | 606 (73%) | 392 (39%) | -- |  |
| Sex (% female) | 135 (61%) | 121 (60%) | 0.85 | 335 (55%) | 205 (52%) | 0.36 |  |
| Race (% White) | 176 (80%) | 165 (82%) | 0.52 | 427 (70%) | 291 (74%) | 0.19 |  |
| **Baseline data** ^a^ | | | | | | | |
| Age (years) |  |  |  |  |  |  |  |
| Visit 1 | 38.9 ± 6.5 | 39.1 ± 6.4 (174) | 0.76 | 11.9 ± 3.2 | 11.7 ± 3.3 (233) | 0.35 |  |
| Visit 2 | 39.7 ± 6.5 | -- | -- | 12.6 ± 3.2 | -- | -- |  |
| Visit 3 | 41.2 ± 7.3 (104) | 41.0 ± 6.9 | 0.86 | 13.8 ± 3.7 (203) | 16.1 ± 6.3 | <0.0001 |  |
| BMI (kg/m^2^) |  |  |  |  |  |  |  |
| Visit 2 | 26.1 ± 4.6 | -- | -- | 19.7 ± 4.4 | -- | -- |  |
| Visit 3 | 25.9 ± 4.4 (88) | 26.4 ± 5.3 (142) | 0.45 | 20.8 ± 5.0 (179) | 21.1 ± 4.5 (269) | 0.60 |  |
| Cholesterol (mmol/L) |  |  |  |  |  |  |  |
| Visit 1 | 5.60 ± 1.10 | 4.83 ± 0.73 (117) | <0.0001 | 4.60 ± 0.95 | 4.19 ± 0.59 (219) | <0.0001 |  |
| Visit 2 | 5.52 ± 1.07 | -- | -- | 4.53 ± 0.88 | -- | -- |  |
| Visit 3 | 5.63 ± 1.16 (104) | 5.28 ± 0.95 | 0.008 | 4.75 ± 1.00 (203) | 4.48 ± 0.78 | 0.0009 |  |
| Triglycerides (mmol/L) |  |  |  |  |  |  |  |
| Visit 1 | 1.78 ± 1.34 | 1.08 ± 0.53 (117) | <0.0001^b^ | 0.96 ± 0.53 | 0.79 ± 0.30 (219) | <0.0001^b^ |  |
| Visit 2 | 1.57 ± 1.04 | -- | -- | 0.86 ± 0.47 | -- | -- |  |
| Visit 3 | 1.54 ± 0.90 (104) | 1.29 ± 0.43 | 0.01^b^ | 1.04 ± 0.85 (203) | 0.95 ± 0.49 | 0.89^b^ |  |
| **Follow-up data** | | | | | | | |
| Age (years) | 66.6 ± 6.5 | 66.2 ± 6.8 | 0.47 | 38.5 ± 3.6 | 41.3 ± 6.1 | <0.0001 |  |
| Marital status (% married) | 158 (76%) | 143 (74%) | 0.94 | 379 (66%) | 233 (61%) | 0.07 |  |
| Education level |  |  |  |  |  |  |  |
| HS grad or less | 107 (49%) | 100 (50%) | 0.39 | 154 (26%) | 105 (27%) | 0.32 |  |
| Some college | 68 (31%) | 56 (28%) |  | 201 (33%) | 142 (37%) |  |  |
| College grad | 32 (15%) | 25 (13%) |  | 165 (27%) | 102 (26%) |  |  |
| Graduate degree | 12 (6%) | 19 (9%) |  | 82 (14%) | 39 (10%) |  |  |
| Annual household income (year 2000 dollars) |  |  |  |  |  |  |  |
| <$10 k | 10 (7%) | 10 (8%) | 0.51 | 4 (2%) | 9 (3%) | 0.48 |  |
| $10-<$25 k | 41 (28%) | 27 (23%) |  | 20 (9%) | 34 (12%) |  |  |
| $25-<$50 k | 54 (36%) | 37 (31%) |  | 59 (26%) | 77 (28%) |  |  |
| $50-<$75 k | 23 (15%) | 20 (17%) |  | 53 (24%) | 66 (24%) |  |  |
| $75 k or more | 21 (14%) | 25 (21%) |  | 87 (39%) | 92 (33%) |  |  |
| BMI (kg/m^2^) | 30.0 ± 6.1 | 29.9 ± 6.5 | 0.89 | 28.6 ± 7.0 | 28.9 ± 6.6 | 0.49 |  |
| Cholesterol (mmol/L) | 5.11 ± 1.09 | 5.02 ± 0.98 | 0.39 | 5.01 ± 1.10 | 4.91 ± 1.07 | 0.17 |  |
| Triglycerides (mmol/L) | 1.86 ± 1.21 | 1.57 ± 0.83 | 0.005^b^ | 1.55 ± 1.61 | 1.54 ± 1.15 | 0.22^b^ |  |
| HDL-C (mmol/L) | 1.19 ± 0.36 | 1.22 ± 0.42 | 0.33 | 1.20 ± 0.40 | 1.13 ± 0.35 | 0.004 |  |
| LDL-C (mmol/L) | 3.07 ± 0.95 | 3.08 ± 0.84 | 0.98 | 3.12 ± 0.95 | 3.08 ± 0.88 | 0.60 |  |
| Glucose (mmol/L) | 5.9 ± 2.2 (209) | 5.8 ± 1.9 (183) | 0.85^b^ | 5.1 ± 1.6 (507) | 5.0 ± 1.7 | 0.50^b^ |  |
| Kcal/day | 1650 ± 779 | 1642 ± 717 | 0.90 | 2051 ± 990 | 1918 ± 966 | 0.04 |  |

Values presented are frequency (percent) or mean ± standard deviation; numbers in parentheses after a continuous variable indicate the number with data. P-values from chi-square tests for categorical data and unpaired t-tests for continuous data, unless otherwise noted.

HS, high school / k, thousand / BMI, body mass index / HDL-C, high density lipoprotein cholesterol / LDL-C, low density lipoprotein cholesterol /Kcal, kilocalories

^a^ Excluded individuals may or may not have attended Visit 1, did not attend Visit 2, and all attended Visit 3 (where blood pressure was not assessed). All included individuals attended Visits 1 and 2 but may or may not have attended Visit 3. Therefore, baseline data are reported for the subsets seen at Visits 1, 2 and 3.

^b^p-values from non-parametric Wilcoxon Rank Sums test due to significant skewness of data

**Supplemental Table 2: Disease outcome cutoff criteria for children**

| **Level** | **Glucose Intolerance** | **High Blood Pressure** | **Dyslipidemia** | **Obesity** |
| --- | --- | --- | --- | --- |
| **Normal** | <6.1 mmol/L | <90^th^ percentile SBP or DBP | TG<1.69 mmol/L  HDL-C>1.03 mmol/L (M)  HDL-C>1.29 mmol/L (F) | <85^th^ percentile BMI |
| **Elevated/ High** | ≥6.1 mmol/L | ≥90^th^ percentile SBP or DBP | TG ≥1.69 mmol/L  HDL-C≤ 1.03 mmol/L (M)  HDL-C≤ 1.29 mmol/L (F) | ≥85^th^ percentile BMI |

M, Male

F, Female

SBP, systolic blood pressure

DBP, diastolic blood pressure

TG, triglycerides

HDL-C, high density lipoprotein cholesterol

**Supplemental Table 3: Disease outcome cutoff criteria for adults**

| **Level** | **Diabetes** | **Hypertension** | **Dyslipidemia** | **Obesity** |
| --- | --- | --- | --- | --- |
| **Normal** | <6.1 mmol/L | <140/90 mmHg | LDL-C ≤3.36 mmol/L  TG ≤1.69 mmol/L  HDL-C>1.03 mmol/L (M)  HDL-C>1.29 mmol/L (F)  Cholesterol <5.17 mmol/L | <25 kg/m^2^ BMI |
| **Moderate to High** | ≥6.1 mmol/L | ≥140/90 mmHg | LDL-C >3.36 mmol/L  TG >1.69 mmol/L  HDL-C ≤1.03 mmol/L (M)  HDL-C ≤1.29 mmol/L (F)  Cholesterol ≥5.17 mmol/L | ≥25 kg/m^2^ BMI |

M, Male

F, Female

LDL-C, low density lipoprotein cholesterol

TG, triglycerides

HDL-C, high density lipoprotein cholesterol

**Supplemental Table 4: Modified nutrient targets for DASH score in children***

|  | **Ages 6 to 10 years^a^** | | **Ages 11 to 13 years^b^** | | **Ages 14 to 19 years^c^** | |
| --- | --- | --- | --- | --- | --- | --- |
| **Nutrient** | **DASH Score Target** | **Intermediate Target** | **DASH Score**  **Target** | **Intermediate**  **Target** | **DASH Score**  **Target** | **Intermediate**  **Target** |
| **Saturated fat** | 6% of energy | 11% of energy | 6% of energy | 11% of energy | 6% of energy | 11% of energy |
| **Total fat** | 27% of energy | 32% of energy | 27% of energy | 32% of energy | 27% of energy | 32% of energy |
| **Protein** | 18% of energy | 16.5% of energy | 18% of energy | 16.5% of energy | 18% of energy | 16.5% of energy |
| **Cholesterol** | 88.2mg/1000kcal | 132.3mg/1000kcal | 75mg/1000kcal | 112.5mg/1000kcal | 63.8mg/1000kcal | 95.7mg/1000kcal |
| **Fiber** | 14.7g/1000kcal | 9.4g/1000kcal | 13g/1000kcal (F) | 8.3g/1000kcal (F) | 11.1g/1000kcal (F) | 7.1g/1000kcal (F) |
|  |  |  | 15.5g/1000kcal (M) | 10g/1000kcal (M) | 16.2/1000kcal (M) | 10.4g/1000kcal (M) |
| **Magnesium** | 141mg/1000kcal | 94mg/1000kcal | 120mg/1000kcal | 80mg/1000kcal | 153mg/1000kcal (F) | 102mg/1000kcal (F) |
|  |  |  |  |  | 174mg/1000kcal (M) | 116mg/1000kcal (M) |
| **Calcium** | 588mg/1000kcal | 401mg/1000kcal | 650mg/1000kcal | 443mg/1000kcal | 553 mg/1000kcal | 377 mg/1000kcal |
| **Potassium** | 2235mg/1000kcal | 1532mg/1000kcal | 2250mg/1000kcal | 1542mg/1000kcal | 2000mg/1000kcal | 1370mg/1000kcal |
| **Sodium** | 1353mg/1000kcal | 1522mg/1000kcal | 1150mg/1000kcal | 1294mg/1000kcal | 979mg/1000kcal | 1101mg/1000kcal |

F, Female

M, Male

^a^Based on a 1,700 kcal diet

^b^Based on a 2,000 kcal diet

^c^Based on a 2,350 kcal diet

*Adapted from *Mellen et al, 2008*

**Supplemental Table 5: Odds ratios and 95% confidence intervals for specific nutrients and disease outcomes at baseline, by generation**

|  | **Baseline Outcomes** | | | |
| --- | --- | --- | --- | --- |
| **Nutrient (Z-score)^a^** | **Diabetes** | **Hypertension** | **Dyslipidemia** | **Obesity** |
| **Parents** |  |  |  |  |
| Protein | 0.90 (0.39, 2.11) | 0.80 (0.56, 1.17) | 0.95 (0.74, 1.23) | **1.41 (1.09, 1.82)** |
| Carbohydrates | **0.28 (0.11, 0.71)** | 1.08 (0.75, 1.56) | 0.75 (0.57, 1.00) | 0.77 (0.58, 1.01) |
| Total fat | **5.67 (1.42, 22.55)** | 0.78 (0.52, 1.15) | 1.26 (0.93, 1.72) | 0.90 (0.67, 1.21) |
| Saturated fat | 2.59 (0.97, 6.91) | 0.76 (0.51, 1.14) | 1.22 (0.91, 1.64) | 0.92 (0.69, 1.23) |
| Cholesterol | 0.89 (0.35, 2.28) | 1.12 (0.79, 1.59) | 1.03 (0.79, 1.35) | 1.15 (0.88, 1.50) |
| Fiber | 0.94 (0.47, 1.88) | 0.87 (0.61, 1.25) | 0.87 (0.67, 1.12) | 0.96 (0.74, 1.24) |
| Sodium | 1.31 (0.46, 3.78) | 0.90 (0.63, 1.27) | 1.20 (0.92, 1.55) | 0.87 (0.66, 1.14) |
| Potassium | 0.97 (0.43, 2.17) | 0.94 (0.67, 1.31) | **1.36 (1.04, 1.76)** | 1.05 (0.81, 1.36) |
| Na:K ratio | 1.07 (0.25, 4.58) | 0.92 (0.54, 1.58) | 0.91 (0.60, 1.39) | 0.76 (0.49, 1.18) |
| Calcium | 1.57 (0.52, 4.69) | 0.84 (0.62, 1.12) | 1.24 (0.89, 1.46) | 1.00 (0.78, 1.28) |
| Iron | 1.40 (0.51, 3.83) | 0.95 (0.66, 1.36) | 1.36 (1.03, 1.80) | 1.18 (0.90, 1.56) |
| Niacin | 0.95 (0.39, 2.28) | 0.96 (0.66, 1.38) | 1.17 (0.88, 1.57) | 1.20 (0.90, 1.60) |
| Vitamin C | 0.73 (0.42, 1.27) | 0.78 (0.56, 1.07) | 1.08 (0.82, 1.42) | 1.09 (0.83, 1.43) |
|  | | | | |
| **Nutrient (Z-score)** | **Diabetes** | **Hypertension** | **Dyslipidemia** | **Obesity** |
| **Offspring** |  |  |  |  |
| Protein | 0.87 (0.24, 3.15) | 1.09 (0.87, 1.36) | 0.99 (0.82, 1.20) | 1.13 (0.86, 1.49) |
| Carbohydrates | 0.41 (0.13, 1.29) | 0.90 (0.73, 1.13) | 1.06 (0.88, 1.28) | 1.16 (0.88, 1.53) |
| Total fat | 2.83 (0.92, 8.68) | 1.09 (0.88, 1.35) | 0.95 (0.79, 1.14) | 0.80 (0.60, 1.05) |
| Saturated fat | 1.57 (0.49, 5.05) | 1.02 (0.82, 1.27) | 1.06 (0.88, 1.28) | **0.70 (0.52, 0.92)** |
| Cholesterol | 2.33 (1.00, 5.43) | 0.87 (0.69, 1.09) | 0.88 (0.72, 1.06) | 0.99 (0.76, 1.28) |
| Fiber | 0.72 (0.18, 2.85) | 1.06 (0.88, 1.29) | 1.06 (0.89, 1.25) | 1.10 (0.88, 1.39) |
| Sodium | **3.95 (1.15, 13.64)** | 0.81 (0.64, 1.02) | 0.96 (0.79, 1.15) | 0.78 (0.58, 1.05) |
| Potassium | 2.16 (0.91, 5.14) | 0.92 (0.73, 1.16) | 1.08 (0.89, 1.30) | 0.77 (0.57, 1.05) |
| Na:K ratio | 1.12 (0.34, 3.63) | 0.79 (0.56, 1.10) | 0.90 (0.69, 1.18) | 0.90 (0.60, 1.33) |
| Calcium | 1.92 (0.77, 4.76) | 0.96 (0.75, 1.22) | 0.96 (0.79, 1.18) | **0.62 (0.43, 0.88)** |
| Iron | 1.76 (0.78, 3.93) | 0.90 (0.72, 1.12) | 1.04 (0.86, 1.25) | 0.90 (0.67, 1.19) |
| Niacin | 1.52 (0.62, 3.73) | 0.87 (0.69, 1.09) | 1.00 (0.83, 1.21) | 1.00 (0.76, 1.31) |
| Vitamin C | 0.45 (0.08, 2.55) | 0.82 (0.65, 1.03) | 1.10 (0.90, 1.28) | 1.00 (0.78, 1.30) |

Odds ratios and 95% confidence intervals (in parentheses) from adjusted logistic regression analyses of categorical disease outcomes at baseline for parents and offspring. Bolded odds ratios represent values for which p<0.05. Adjustments were made for sex, race, age, and body mass index where obesity was not the outcome measured.

Na:K ratio: sodium to potassium ratio

^a^Age and sex-specific z-scores of nutrient intake, adjusted for total energy intake

**Supplemental Table 6: Odds ratios and 95% confidence intervals for specific nutrients and disease outcomes at follow-up, by generation**

|  | **Follow-up Outcomes** | | | |
| --- | --- | --- | --- | --- |
| **Nutrient (Z-score)^a^** | **Diabetes** | **Hypertension** | **Dyslipidemia** | **Obesity** |
| **Parents** |  |  |  |  |
| Protein | 0.98 (0.70, 1.37) | 1.22 (0.91, 1.65) | 1.12 (0.75, 1.68) | 1.02 (0.72, 1.45) |
| Carbohydrates | 0.79 (0.57, 1.10) | 0.81 (0.61, 1.08) | 1.02 (0.69, 1.50) | 0.99 (0.71, 1.37) |
| Total fat | **1.54 (1.07, 2.23)** | 1.16 (0.86, 1.57) | 1.19 (0.80, 1.79) | 1.06 (0.76, 1.49) |
| Saturated fat | **1.63 (1.12, 2.37)** | 1.15 (0.84, 1.56) | 1.38 (0.91, 2.09) | 0.94 (0.66, 1.33) |
| Cholesterol | 1.26 (0.91, 1.73) | 1.09 (0.80, 1.48) | 0.95 (0.65, 1.39) | 0.97 (0.70, 1.36) |
| Fiber | 0.91 (0.64, 1.28) | 0.81 (0.60, 1.11) | 0.99 (0.67, 1.45) | 1.15 (0.81, 1.65) |
| Sodium | 1.01 (0.69, 1.45) | 1.19 (0.86, 1.66) | 1.09 (0.70, 1.70) | 0.97 (0.68, 1.38) |
| Potassium | 1.01 (0.71, 1.42) | 0.94 (0.69, 1.28) | 0.91 (0.61, 1.37) | 1.11 (0.78, 1.60) |
| Na:K ratio | 1.06 (0.76, 1.48) | 1.19 (0.88, 1.62) | 1.10 (0.75, 1.60) | 0.91 (0.65, 1.28) |
| Calcium | 1.05 (0.74, 1.47) | 1.04 (0.77, 1.39) | 1.12 (0.72, 1.72) | 0.93 (0.67, 1.30) |
| Iron | 0.63 (0.40, 1.00) | 0.93 (0.70, 1.22) | 1.05 (0.70, 1.59) | 1.17 (0.82, 1.66) |
| Niacin | **0.59 (0.38, 0.90)** | 1.11 (0.83, 1.48) | 1.12 (0.74, 1.72) | 0.99 (0.71, 1.38) |
| Vitamin C | 0.99 (0.72, 1.35) | 0.83 (0.62, 1.11) | 0.82 (0.58, 1.17) | 1.11 (0.79, 1.55) |
|  | | | | |
| **Nutrient (Z-score)** | **Diabetes** | **Hypertension** | **Dyslipidemia** | **Obesity** |
| **Offspring** |  |  |  |  |
| Protein | **1.62 (1.13, 2.33)** | **0.78 (0.62, 0.99)** | 0.83 (0.68, 1.02) | 1.14 (0.95, 1.37) |
| Carbohydrates | 0.76 (0.52, 1.12) | 0.85 (0.68, 1.07) | 1.01 (0.82, 1.24) | 0.95 (0.79, 1.13) |
| Total fat | 1.02 (0.71, 1.47) | 1.15 (0.92, 1.44) | 1.17 (0.95, 1.43) | 1.06 (0.89, 1.27) |
| Saturated fat | 1.09 (0.75, 1.60) | 1.16 (0.92, 1.45) | 1.12 (0.91, 1.37) | 1.05 (0.88, 1.25) |
| Cholesterol | 1.25 (0.90, 1.73) | 0.92 (0.76, 1.17) | 0.91 (0.73, 1.13) | 1.19 (0.99, 1.44) |
| Fiber | 0.96 (0.66, 1.38) | 0.80 (0.63, 1.02) | **0.74 (0.61, 0.90)** | 0.89 (0.75, 1.05) |
| Sodium | 1.05 (0.73, 1.53) | 0.88 (0.70, 1.11) | 0.85 (0.69, 1.05) | 0.92 (0.77, 1.10) |
| Potassium | 1.05 (0.71, 1.54) | **0.75 (0.58, 0.97)** | **0.79 (0.65, 0.98)** | 0.94 (0.78, 1.12) |
| Na:K ratio | 0.91 (0.63, 1.31) | 1.20 (0.96, 1.51) | 1.07 (0.87, 1.33) | 0.98 (0.82, 1.17) |
| Calcium | 1.23 (0.86, 1.78) | 0.95 (0.75, 1.21) | 0.96 (0.78, 1.18) | 0.93 (0.78, 1.12) |
| Iron | 0.92 (0.61, 1.40) | **0.70 (0.53, 0.92)** | 0.85 (0.69, 1.04) | 0.95 (0.79, 1.14) |
| Niacin | 1.26 (0.89, 1.79) | **0.74 (0.58, 0.94)** | **0.80 (0.66, 0.98)** | 1.02 (0.85, 1.21) |
| Vitamin C | 0.85 (0.57, 1.25) | 0.98 (0.78, 1.22) | 0.91 (0.74, 1.12) | 1.06 (0.88, 1.28) |

Odds ratios and 95% confidence intervals (in parentheses) from adjusted logistic regression analyses of categorical disease outcomes at follow-up for parents and offspring. Bolded odds ratios represent values for which p<0.05. Adjustments were made for sex, race, age, and body mass index where obesity was not the outcome measured.

Na:K ratio: sodium to potassium ratio

^a^Age and sex-specific z-scores of nutrient intake, adjusted for total energy intake
